# Identification, Molecular Characterization, and Biology of a Novel *Quadrivirus* Infecting the Plant Fungus *Allocryptovalsa sichuanensis*

**DOI:** 10.3390/v17020275

**Published:** 2025-02-17

**Authors:** Yuxu Xie, Xianhong Wang, Xiuyuan Sun, Fanxing Ren, Meng Zhang, Chao Xu, Qingzhou Ma, Yuehua Geng, Rui Zang, Yashuang Guo

**Affiliations:** 1 College of Plant Protection, Henan Agricultural University, Zhengzhou 450002, China; xyx6858@163.com (Y.X.); xysun1225@163.com (X.S.); r3197370145@163.com (F.R.); zm2006@126.com (M.Z.); chaoxu01@163.com (C.X.); 15237171177@163.com (Q.M.); gengyuehua@163.com (Y.G.); zangrui@henau.edu.cn (R.Z.); 2 Key Lab of Plant Pathology of Hubei Province, College of Plant Science and Technology, Huazhong Agricultural University, Wuhan 430070, China; xianhong@webmail.hzau.edu.cn

**Keywords:** *Allocryptovalsa sichuanensis*, *Allocryptovalsa sichuanensis* quadrivirus 1, dsRNA mycoviruses, *Quadriviridae*

## Abstract

A novel double-stranded RNA (dsRNA) virus was isolated and described from strain ZZZ210557 of plant endophyte *Allocryptovalsa sichuanensis*. The dsRNA virus contains four dsRNA segments, dsRNA1 to dsRNA4, with a size range of 3.8 to 5.1 kbp. Each possesses a single large ORF and is encapsulated in isometric particles approximately 42–47 nm in diameter. Notably, the dsRNA3 encoded sequence revealed modest similarities to the amino acid sequences of RdRps predicted from the nucleotide sequences of known and suspected members of the family *Quadriviridae*. Phylogenetic analysis of the putative RdRp with the corresponding proteins of other quadriviruses revealed that the dsRNA virus is a new member belonging to the family *Quadriviridae*, tentatively named *Allocryptovalsa sichuanensis* quadrivirus 1 (AsQV1). All four segments of AsQV1 could be successfully cured through ribavirin treatment, whereas it likely has no apparent impact on the morphologies or virulence of the host fungus. This study is the first report of a quadrivirus isolated from the fungus *A. sichuanensis*, and our results enhance the diversity of the quadrivirus.

## 1. Introduction

Mycoviruses (fungal viruses) have been widely reported in all major taxa of fungi, including filamentous fungi, yeasts, and oomycetes [1,2,3,4,5,6]. Based on the nature of their genomic nucleic acids, mycoviruses have been assigned to 42 families, 83 genera, and 534 approved species by the International Committee on Taxonomy of Viruses (ICTV, https://talk.ictvonline.org, accessed on 18 December 2023), with some mycoviruses remaining unclassified at the family or genus level [7]. Most of the mycoviruses’ genomes are double-stranded RNA (dsRNA) or positive-sense single-stranded RNA (+ssRNA) [8,9], and a few are negative-sense single-stranded RNA (-ssRNA) [10] or single-stranded circular DNA (ssDNA) [11].

The family *Quadriviridae*, which was established in 2013, at present, only includes the single genus *Quadrivirus* with three species (*Quadrivirus ichi*, *Quadrivirus ni*, and *Quadrivirus sani*). The *Quadriviridae* is a monogeneric family of non-enveloped spherical viruses with quadripartite dsRNA genomes, termed dsRNA1-4, each approximately 3.5–5.0 kbp and possessing a single large ORF [12]. The function of the dsRNA1 translation product remains unknown, but both dsRNA2 and dsRNA4 encode capsid proteins (CPs) that co-assemble capsids, and dsRNA3 encodes RNA-dependent RNA polymerase (RdRp) [13,14].

The *Diatrypaceae*, a family of fungi, remained relatively obscure until recent advancements in 2020, when extensive revisions and expansions led to the recognition of over 20 genera within this family [15]. Based on this revised taxonomy, Senwanna et al. (2017) introduced *Allocryptovalsa*, with *Allocryptovalsa polyspora* as its designated type species [16]. *Allocryptovalsa* fungi live in host plants with a complex lifestyle, being parasitic or commensal. *Allocryptovalsa sichuanensis* was isolated and identified by M.C. Samarakoon in 2019 from a dead branch at the University of Electronic Science and Technology of China (Qingshuihe Campus) in Chengdu, Sichuan Province, China [16,17,18,19]. Mycoviruses might have participated in the transition of these lifestyles; for example, *Sclerotinia sclerotiorum* strain DT-8 infected by SsHADV1 lost its ability to cause disease in plants but could still penetrate plant cell walls and grow inside the plant as endophytes to trigger plant defense and hormone signaling genes, promoting plant growth and enhancing immunity [20,21]. Since the early 1950s, when a hypovirulent strain of the chestnut blight fungus (*Cryphonectria parasitica*) was isolated from a self-curing canker of a chestnut tree and was demonstrated to potentially control chestnut blight [22,23,24], the characterization of a hypovirus in fungal species became of great interest. In this study, we attempted to detect mycoviruses in *A. sichuanensis*, an endophytic fungus isolated from jujube trees, and a novel dsRNA virus from strain ZZZ210557 was identified and characterized. This is the first quadrivirus discovered in *A. sichuanensis*, tentatively named *Allocryptovalsa sichuanensis* quadrivirus 1 (AsQV1). We conducted a complete analysis of the genome structure and phylogeny of the virus and described in detail the effects of the virus on host phenotype and pathogenicity.

## 2. Materials and Methods

### 2.1. Fungal Strains and Cultures

*A. sichuanensis* strain ZZZ210557 was isolated from jujube tree branches showing shoot canker symptoms collected in Zhengzhou, Henan Province, China (113.664° E, 34.784° N), and was identified based on morphologies and multi-locus sequences (ITS, *rpb2*, *SSU*, *tef1*, and *tub2*) as previously described [25,26,27,28]. Five virus-free strains, ZZZ210557-1, ZZZ210557-2, ZZZ210557-3, ZZZ210557-4, and ZZZ210557-5, are subisolates generated by ribavirin treatment to eliminate the mycoviruses from ZZZ210557 [29]. All strains were cultured at 25 °C in the dark for 3–5 days on potato dextrose agar (PDA) plates and stored in sterile 30% glycerol solution at 4 °C.

### 2.2. dsRNA Extraction, Purification, and Enzymatic Treatments

For dsRNA extraction, fungal mycelial plugs were inoculated onto cellophane membranes overlaid on PDA plates at 25 °C in the dark for 5 days, and the mycelia were collected and subjected to dsRNA extraction as previously described [30]. The resulting nucleic acids were treated with DNase I (RNase-free) and S1 nuclease (TaKaRa, Dalian, China) to eliminate any contaminating DNA and ssRNA. The purified dsRNA samples were electrophoresed in a 1.0% (*w*/*v*) agarose gel stained with Goldview and viewed on a UV transilluminator.

### 2.3. cDNA Synthesis and Molecular Cloning

The total RNA (1.0 μg) was used as a template to efficiently reverse the full-length cDNA using the HiScript-TS 5′/3′ RACE Kit (Vazyme, Nanjing, China) according to the manufacturer’s instructions. The terminal sequences of AsQV1 were obtained using nested primers designed based on the central sequences of AsQV1 and ligase-mediated rapid amplification of cDNA ends (RLM-RACE) [31]. The amplified products were ligated into the pMD18-T vector (TaKaRa, Dalian, China) and then transformed into competent cells of Escherichia coli DH5α. Sequence gaps between clones were determined by reverse transcription–polymerase chain reaction (RT-PCR) using primers designed from the obtained cDNA sequences. In addition, sequencing was performed by Sangon Biotech Company, Ltd. (Shanghai, China). At least three independent positive recombinant plasmids of each product were sequenced in both directions to ensure the accuracy of the sequence. All primers used for molecular cloning are listed in Supplemental Table S1.

### 2.4. Sequence Alignment and Phylogenetic Analysis

Searches of sequence similarity were performed using the BLASTx or BLASTp program on the NCBI website (https://blast.ncbi.nlm.nih.gov/Blast.cgi, accessed on 20 November 2023). The final viral genome sequences were assembled using DNAMAN software (version 5.2.2). The open reading frames (ORFs) were predicted using the ORFfinder tool on the NCBI website (https://www.ncbi.nlm.nih.gov/orffinder/, accessed on 6 January 2024). The phylogenetic tree was constructed using MEGA 7.0 software by the maximum-likelihood (ML) method with default parameters (Poisson model) and a bootstrap test consisting of 1000 replicates.

### 2.5. Eliminate Viruses from Strains

It has been reported that mycoviruses in strains can be eliminated by protoplast regeneration, hyphal tipping, and ribavirin treatment [29]. Due to the fact that sporulation was induced in strain ZZZ210557 without success, we attempted to eliminate mycoviruses with ribavirin treatment. The strain ZZZ210557 was cultured on PDA medium supplemented with 100 μg/mL ribavirin (Solarbio, Beijing, China), and subisolates of strain ZZZ210557 obtained using hyphal tips 3–5 mm 10 times were detected by RT-PCR amplification and dsRNA extraction.

### 2.6. Purification and Visualization of Virus Particles

Virus particles from mycelia were purified by sucrose density gradient centrifugation as previously described [32]. Sucrose fractions were carefully collected, loaded onto carbon-coated 230-mesh copper grids, negatively stained with 2% (*w*/*v*) uranyl acetate, and observed under a transmission electron microscope (H7650 and H-7000FA; Hitachi, Japan).

### 2.7. Growth Rate, Morphology, and Virulence Assays

Fungal growth rates and morphologies were assessed as previously described [33]. Briefly, mycelial discs (5 mm in diameter) from fresh mycelia were transferred onto PDA and incubated in darkness at 25 °C. Colony diameter was measured daily for 3 days to calculate growth rates (millimeters per day). Fungal virulence was determined on detached jujube leaves, branches (*Ziziphus* sp.), and fruits (*Zizyphus mauritiana* Lam.) as previously described [33]. The inoculated materials were cultured in the greenhouse at 28 °C and 90% relative humidity with a 12 h/12 h photoperiod. Four replicates of two independent experiments were used.

## 3. Results

### 3.1. Double-Stranded RNA Segments Were Identified in A. sichuanensis

**Figure 1:**
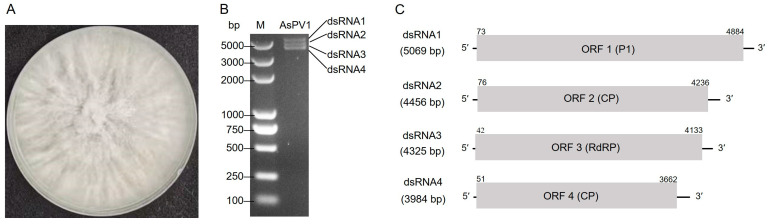
The colonial morphology, dsRNA genome, and genomic organization of AsQV1. (**A**) Colony morphology of *A. sichuanensis* strain ZZZ210557 cultured on PDA medium (25 °C, 10 days); (**B**) Electrophoresis analysis of dsRNA extracted from strain ZZZ210557 on agarose gel; Lane M, DL5000 DNA marker, the sizes of which are shown to the left of the gel. (**C**) Genomic organization of AsQV1 dsRNAs 1–4 showing putative ORFs and untranslated regions (UTRs).

**Figure 2:**
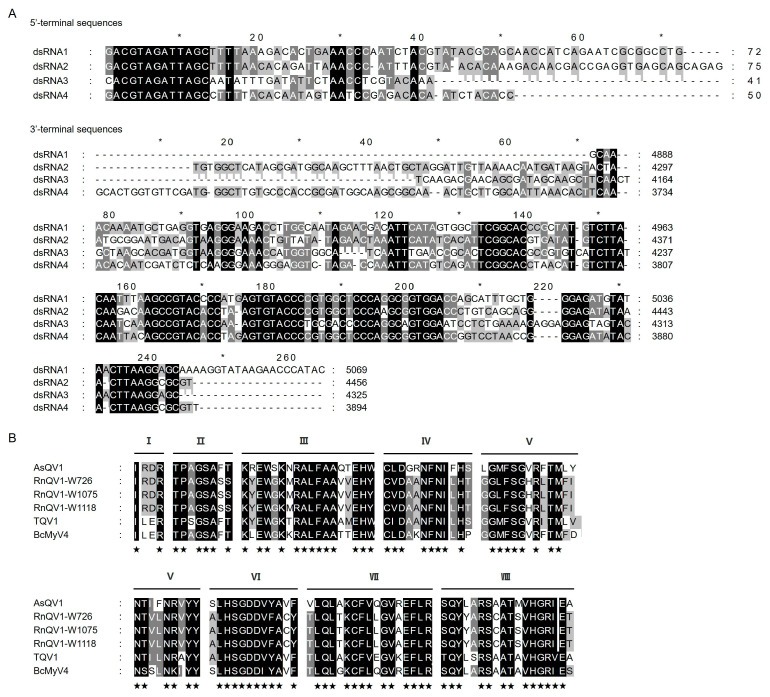
Multiple sequence alignment of 5′- and 3′-UTRs and RdRp amino acid sequences. (**A**) Conserved sequences of the 5′- and 3′-termini of the dsRNAs, respectively. Identical sequences are denoted by asterisks. (**B**) Sequence alignment of AsQV1 RdRp motifs with those of selected members of the family *Quadriviridae*. The horizontal black lines above the sequence alignment represent the eight conserved motifs (I–VIII). The shaded areas indicate identical amino acid residues. ★, conserved amino acid residues.

Nucleic acid preparations enriched in dsRNA were obtained from *A. sichuanensis* strain ZZZ210557 isolated from jujube tree branches collected in China (Figure 1A) and were subjected to digestion with DNase I and S1 nuclease and then agarose gel electrophoresis. The results showed that four dsRNAs (nominated 1–4 according to their decreasing sizes) were detected in preparations of strain ZZZ210557 (Figure 1B). Following RT-PCR with tagged random primers and RLM-RACE, the sequences of the full-length cDNA clones derived from dsRNAs 1 to 4 were determined. The lengths of dsRNAs 1 to 4 were 5069, 4456, 4325, and 3894 bp, respectively (Figure 1C). All RNAs contained one ORF (serially termed ORF1 to ORF4) flanked by untranslated regions (UTRs) and encoded four putative proteins (termed P1 to P4). The 5′-UTRs of the coding strands of dsRNAs 1 to 4 ranged from 41 to 75 bp in size (Figure 1C). All contained a highly conserved 12 bp sequence (ACGTAGATTAGC) at their 5′-termini (Figure 2A). The corresponding 3′-UTRs ranged from 185 to 232 bp in size (Figure 1C) and contained conserved nucleotides at the 3′-termini of dsRNAs 1 to 4 (Figure 2A). The corresponding sequences were deposited in GenBank with accession numbers PQ683691-PQ683694.

### 3.2. Analysis of the Putative Proteins Encoded by the dsRNA ORFs

All putative proteins encoded by ORFs 1 to 4 (termed P1 to P4), ranging from 1203 to 1603 amino acids (aa) in size, were assessed for the presence of conserved domains using the National Center for Biotechnology Information (NCBI) Conserved Domain (CD) search tool and similarity with known proteins in the NCBI database using the Basic Local Alignment Search Tool (BLASTp).

P1 is encoded by dsRNA1, coding for a protein of 1603 aa with an approximate molecular mass of 178.6 kDa, which was the most similar to the uncharacterized protein of Leptosphaeria biglobosa quadrivirus 1 (LbQV-1, GenBank accession numberVCV25421, 96% coverage, 29.15% identity, E value = 0.0). P2 is encoded by dsRNA2, coding for a protein of 1386 aa with an approximate molecular mass of 151.2 kDa, which was the most similar to the putative capsid protein of LbQV-1 (VCV25422, 99% coverage, 40.03% identity, E value = 0.0). P3 is encoded by dsRNA3 and coded for a protein of 1363 aa with an approximate molecular mass of 151.3 kDa. This protein was the most similar to the RdRp of LbQV-1 (VCV25423, 99% coverage, 50.89% identity, E value = 0.0). P4 is encoded by dsRNA4 and coded for a protein of 1203 aa with an approximate molecular mass of 129.6 kDa. This protein was the most similar to the putative capsid protein of LbQV-1 (VCV25424, 86% coverage, 39.14% identity, E value = 0.0) (Supplemental Table S2).

### 3.3. Phylogenetic Analysis of the RdRp Encoded by dsRNA3 and Related Proteins

**Figure 3:**
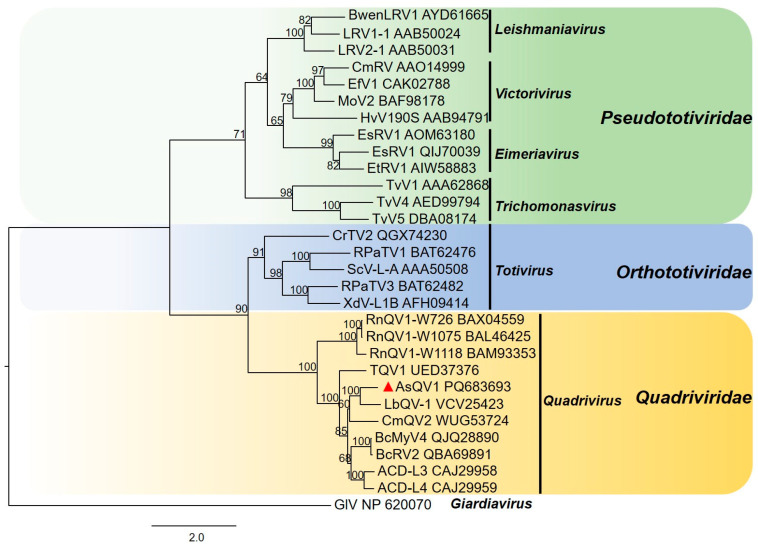
Phylogenetic analysis based on RdRps of AsQV1 and closely related members selected from each genus of the families *Quadriviridae* and *Totiviridae*. Numbers at the nodes indicate bootstrap values out of 1000 replicates (only values > 50 are shown). The position of AsQV1 is marked with a red triangle.

Multiple sequence alignment based on the amino acid sequence of the RdRp domains of AsQV1 and other quadriviruses revealed eight highly conserved motifs I–VIII (Figure 2B). A phylogenetic tree was constructed based on P3 and LbQV-1 RdRp, together with RdRps from exemplars belonging to the *Quadriviridae* and dsRNA virus families (*Orthototiviridae* and *Pseudototiviridae*). As shown in Figure 3, P3 together with the RdRps encoded by LbQV-1, LbQV-1, BcRV2, ACD-L3, and ACD-L4 formed a distinct clade in the *Quadriviridae* family.

Based on their molecular characteristics, genomic organization, similarity to other viruses, and phylogenetic analysis, these dsRNAs are proposed as a novel species in the *Quadriviridae* family, tentatively named *Allocryptovalsa sichuanensis* quadrivirus 1 (AsQV1).

### 3.4. Viral Particles of AsQV1

**Figure 4:**
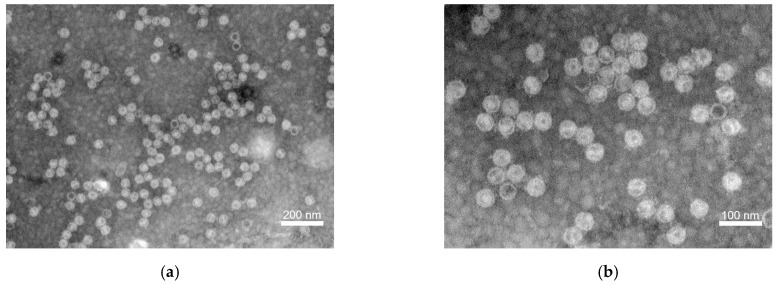
Viral particles of AsQV1 purified from ZZZ210557 mycelia as visualized by TEM. (**a**) Size bars indicate 200 nm. (**b**) Size bars indicate 100 nm.

To isolate AsQV1 particles, ZZZ210557 mycelia were subjected to virion purification using sucrose density gradient ultracentrifugation (10–50% [*w*/*v*], with increments of 10%). Transmission electron microscopy (TEM) revealed isometric particles with a diameter of 42–47 nm in the 30% sucrose fractions (Figure 4).

### 3.5. Effects of AsQV1 on A. sichuanensis

**Figure 5:**
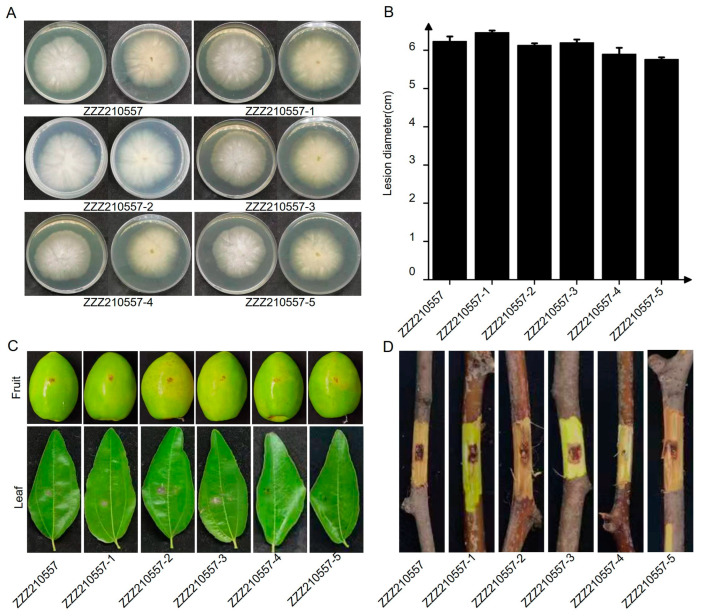
Biological characteristic comparison between virus-infected strains and virus-free strains. (**A**) Colony morphology on PDA medium (25 °C, 5 days). (**B**) Statistical analysis of the growth rate. (**C**) Jujube fruits and leaves wound-inoculated with colonized plugs of tested strains and photographed at 4 dpi. (**D**) Jujube branches wound-inoculated with colonized plugs of tested strains and photographed at 7 dpi.

To investigate the biological traits of AsQV1, attempts were made to obtain an AsQV1-free isogenic line by single conidia isolation. Sporulation was induced in strain ZZZ210557 using several approaches as previously described [34] but without success. Meanwhile, the attempt to eliminate AsQV1 in strain ZZZ210557 following treatment with 100 μg/mL ribavirin obtained five subisolates (ZZZ210557-1, ZZZ210557-2, ZZZ210557-3, ZZZ210557-4, and ZZZ210557-5) without AsQV1 successfully. Compared with the strain ZZZ210557, the colonial morphology of the subisolates (ZZZ210557-1, ZZZ210557-2, ZZZ210557-3, ZZZ210557-4, and ZZZ210557-5) was not significantly different (Figure 5A). Additionally, the growth rates on PDA of the subisolates (ZZZ210557-1, ZZZ210557-2, ZZZ210557-3, ZZZ210557-4, and ZZZ210557-5) did not significantly differ from that of strain ZZZ210557 (15.7–16.5 mm/day vs. 16.3 mm/day) (Figure 5B).

At the same time, all tested strains induced similar-sized lesions when inoculated on jujube fruits, leaves (Figure 5C), and branches (Figure 5D). Therefore, these results suggest that AsQV1 confers no obvious effects on the morphologies or virulence of *A. sichuanensis*.

## 4. Discussion

In this study, four dsRNA components were detected in *A. sichuanensis*, and their full-length sequences were determined and characterized, suggesting that they belong to a novel quadrivirus, tentatively named AsQV1, under the family *Quadriviridae*. The *Quadriviridae* is a monogeneric family with a multipartite genome consisting of four monocistronic dsRNA segments (genome sizes range from 3.5 to 5.0 kbp), in which one or two dsRNA segments are encapsidated in a similar particle [12,35]. Whereas most dsRNA virus capsids are based on the dimers of a single protein, Rosellinia necatrix quadrivirus 1 (RnQV1) has a single-shelled T = 1 capsid formed by 60 heterodimers of two proteins of 1356 (P2) and 1059 residues (P4) [36]. In the same way, AsQV1 contains two major structural proteins P2 and P4 encoded by dsRNA2 and dsRNA4, respectively, which form spherical virus particles with a diameter of about 40–45 nm (Figure 4).

A significant characteristic of mycoviruses is their exclusive reliance on intracellular transmission mechanisms, as they lack extracellular dissemination pathways [2]. These mycoviruses primarily propagate through vertical transmission via sporogenesis or horizontal transfer through hyphal anastomosis [37]. Previous studies have documented quadrivirus infections across diverse fungal taxa, including *Rosellinia necatrix*, *Thelonectria* sp., *Leptosphaeria biglobosa*, and *Botrytis ciner* [13,38,39], consistent with the broader observation of mycovirus cross-species infectivity among different fungal genera in natural ecosystems. To investigate horizontal transmission potential, we conducted pairwise co-cultivation experiments using AsQV1-infected strain ZZZ210557 as the donor and 21 virus-free recipient strains representing five phytopathogenic genera (*Botryosphaeria*, *Lasiodiplodia*, *Cytospora*, *Diaporthe,* and *Diplodia*). Despite systematic attempts, no successful horizontal transfer was detected under experimental conditions, suggesting that AsQV1 may require specific transmission determinants that warrant further investigation.

Unlike LbQV-1, which elicits alterations to the growth and pathogenicity of *L. biglobosa* resulting in the uncommon occurrence of hypervirulence [39], AsQV1 confers no obvious effects on the morphologies or virulence of *A. sichuanensis* (Figure 5). *L. biglobosa* was isolated and identified from *Brassica napus*. Previous studies have shown that *B. napus* inoculated with LbQV-1 contains the highest relative percentage of fungal reads. Analysis of the plant transcriptome revealed that the presence of the virus leads to subtle alterations in metabolism and plant defense, ultimately resulting in hypervirulence. Subsequently, we can also investigate the mechanism of action of AsQV1 from this perspective [40].

Mycoviruses may have diverse ecological functions in various fungal hosts; for example, Leptosphaeria biglobosa botybirnavirus 1 does not exhibit any noticeable effects on *L. biglobosa*, but it induces hypovirulence in its new fungal host *B. cinerea* [41]. Despite the often asymptomatic nature of mycovirus infections, ostensibly asymptomatic infections may still influence host physiology and phenotypes under stressful conditions [42]. Different factors like temperature, growth media, fungal hosts, or mycoviruses have the potential to augment, diminish, or reverse phenotypic outcomes caused by mycoviruses [43]. It is not known whether AsQV1 will have an impact on host fungi in more ways or under different environmental factors.

## Data Availability

The original contributions presented in this study are included in the article and Supplementary Materials. Further inquiries can be directed to the corresponding authors.

## Supplementary Materials

The following supporting information can be downloaded at: https://www.mdpi.com/article/10.3390/v17020275/s1, Table S1: List of primers used in this study; Table S2: Results of BLASTp search with AsQV1 genomic segments.
